# Unusual Manifestations of Monoclonal Gammopathy: I. Ocular Disease

**DOI:** 10.5041/RMMJ.10211

**Published:** 2015-07-30

**Authors:** Sophia R. Balderman, Marshall A. Lichtman

**Affiliations:** 1 Instructor in Medicine, James P. Wilmot Cancer Center, University of Rochester Medical Center, Rochester, NY, USA; 2 Professor of Medicine and of Biochemistry and Biophysics, James P. Wilmot Cancer Center, University of Rochester Medical Center, Rochester, NY, USA

**Keywords:** Copper deposition, crystal-storing histiocytosis, crystalline keratopathy, monoclonal gammopathy, monoclonal gammopathy of undetermined significance (MGUS), ocular disorders

## Abstract

Essential monoclonal gammopathy is usually an asymptomatic condition, the characteristics of which have been defined over approximately 70 years of study. It has a known population-attributable risk of undergoing clonal evolution to a progressive, symptomatic B-cell neoplasm. In a very small fraction of patients, the monoclonal immunoglobulin has biophysical characteristics that can lead to tissue deposition syndrome (e.g. Fanconi renal syndrome) or, by chance, have characteristics of an autoantibody that may inactivate critical proteins (e.g. acquired von Willebrand disease). In this report, we describe the very uncommon forms of ocular injury that may accompany essential monoclonal gammopathy, which include crystalline keratopathy, crystal-storing histiocytosis, hypercupremic keratopathy, and maculopathy. The first three syndromes result from uncommon physicochemical alterations of the monoclonal immunoglobulin that favor crystallization or exaggerated copper binding. The last-mentioned syndrome is of uncertain pathogenesis. These syndromes may result in decreased visual acuity. These ocular findings may lead, also, to the diagnosis of monoclonal gammopathy.

## INTRODUCTION

Essential monoclonal gammopathy (synonymous with monoclonal gammopathy of unknown significance) has a known population-attributable risk of progressing from a stable clone, characteristically with no apparent health consequences, to a progressive B-lymphocytic neoplasm in approximately 1% of affected persons per year.^1^,^2^ Uncommonly, the monoclonal immunoglobulin may act to injure tissue by immunoglobulin deposition disease (e.g. Fanconi renal syndrome) or by acting as an autoantibody and impairing the function of a specific protein (e.g. acquired von Willebrand disease). These uncommon events may occur in any form of monoclonal gammopathy ranging from essential monoclonal gammopathy to a B-cell malignancy (e.g. lymphoma, macroglobulinemia, myeloma, or amyloidosis).

Ophthalmic injury is a rare consequence of monoclonal gammopathy. Monoclonal gammopathy can induce pathological changes in the eye (and surrounding tissue) that can lead to loss of visual acuity or compromised visual function. Awareness of this relationship is important for two reasons: (1) patients who develop ocular pathology of the types described herein should have appropriate studies of serum immunoglobulins since in cases of severe ocular disease treatment of the ophthalmological injury and of the monoclonal gammopathy may be warranted; and, (2) physicians following patients with monoclonal gammopathy should be aware of the possibility of development of associated ocular disease, so as to intervene, if possible, before damage is advanced. In this report we highlight the major types of ocular disorders associated with essential monoclonal gammopathy.

In the earliest reported cases of ocular disorders caused by a monoclonal protein, the patient’s clinical findings usually were indicative of myeloma or another established B-cell malignancy, although in some cases the patient’s history indicated that the ocular pathology preceded the development of the B-cell malignancy, usually myeloma. We can understand this observation today, as evidence indicates that virtually all cases of myeloma are preceded by a period of essential monoclonal gammopathy; the stable clone had not at that time acquired the additional cooperating somatic mutations necessary to evolve into a B-cell malignancy. Since the ocular pathology is related to the physicochemical peculiarities of the monoclonal immunoglobulin (e.g. predisposition to form crystals) rather than to its concentration in the plasma, and it is unlikely that that feature changes when the monoclonal gammopathy undergoes clonal evolution to a progressive B-cell malignancy (e.g. myeloma), these phenomena when diagnosed at the later myeloma stage may have been present at the time of the earlier essential monoclonal gammopathy stage. Since the ocular alterations may be asymptomatic, their discovery might await the intensive evaluation that accompanies the diagnosis of myeloma or another B-cell malignancy. If the ocular findings are symptomatic, the ophthalmologist would have to consider and explore the possibility of an accompanying non-progressive monoclonal gammopathy. In occasional cases, the ocular disorder results in the diagnosis of essential monoclonal gammopathy or a B-cell malignancy (e.g. myeloma).

The critical variable in the ocular disorder is the physicochemical property of the monoclonal immunoglobulin (e.g. tendency to immunoglobulin crystallization, high affinity for copper). These characteristics are present in the patient at the time that essential monoclonal gammopathy occurs, and these effects of the monoclonal immunoglobulin are independent of the progression of the clone to a symptomatic B-cell neoplasm. We focus on cases that at the time of the diagnosis of the ocular disorder were characteristic of essential monoclonal gammopathy.

## CRYSTALLINE KERATOPATHY

The earliest descriptions of keratopathy that relate to a disease associated with monoclonal gammopathy (e.g. myeloma) date to the 1930s as described by the German ophthalmologist Meesman.^3^ He presciently postulated that the ocular injury was related to Bence Jones protein. Burki in 1953 reported a patient with keratopathy, very likely accompanying essential monoclonal gammopathy; three-and-a-half years later the patient developed myeloma.^4^,^5^ The association of keratopathy with an uncommon crystallizing monoclonal immunoglobulin had to await the translation of Arne Tiselius’s ground-breaking basic studies on the separation of proteins in liquid phase into practical techniques, such as zonal electrophoresis and immunoelectrophoresis of serum, applicable to the medical clinic. The application of serum protein separation to medical diagnosis and the more advanced understanding of the physicochemical structural and functional features of polyclonal and monoclonal immunoglobulins led to their firm association with the keratopathic syndromes.

**Figure 1:**
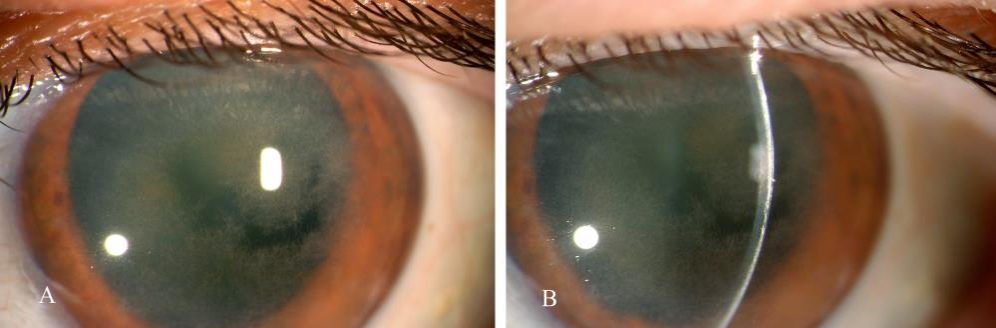
Patient with IgG-kappa Essential Monoclonal Gammopathy In 1999, a 64-year-old woman presented to the University of Rochester, Department of Ophthalmology, with diminished visual acuity, bilaterally. (A) Ocular examination showed widely dispersed straw-like interlacing deposits in the corneas of both eyes, strongly suggesting crystalline keratopathy. In this frontal view, the irregular dark areas represent more normal corneal light transmission in relatively unaffected areas centrally. Most of the cornea contains a network of apparent crystals forming an interlacing opacity. (B) Slit lamp examination showed the deposits were in the anterior corneal stroma resulting in an arc of opaque white coloration. The patient had no family history of corneal dystrophies. Crystalline keratopathy related to a crystal-forming monoclonal Ig was suspected. Indeed, she had a 0.5 g/dL serum monoclonal immunoglobulin, IgG-kappa, by zonal and immunoelectrophoresis. She has had no findings to indicate a progressive B-cell neoplasm during 16 years of follow-up. She had bilateral penetrating keratoplasty with restoration of visual acuity. (The authors thank Holly B. Hindman, M.D., University of Rochester, Department of Ophthalmology, for providing these images.)

**Figure 2:**
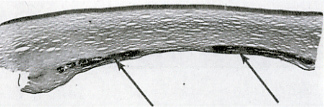
Patient with IgG-kappa Essential Monoclonal Gammopathy Corneal button from a penetrating keratoplasty was prepared for histological examination. This image shows a normal epithelium and anterior and mid–stromal layers. The arrows point to immunoglobulin deposits in the posterior stroma adjacent to Descemet membrane (see Figure 3). (Used from reference 7 with permission of *Archives of Ophthalmology*.)

**Figure 3:**
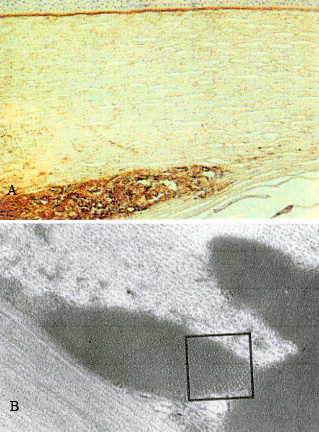
Patient with IgG-kappa Essential Monoclonal Gammopathy Corneal button from a penetrating keratoplasty was prepared for histological examination. (A) This image is in the same area as the image in Figure 2 and indicates the deposition of Ig composed of kappa light chains by the immunoperoxidase reaction (brown stain). (B) Transmission electron microscopy uncovered electron-dense extracellular deposits in the same area that exhibited a honeycomb pattern or parallel linear structures with a periodicity of 10–11 nm (original magnification ×27,000). (Used from reference 7 with permission of *Archives of Ophthalmology*.)

In 1978, the association of corneal crystals with a serum monoclonal IgG-kappa in a case of myeloma was reported,^6^ and shortly thereafter the same relationship was observed in a patient with essential monoclonal gammopathy.^7^ Investigation of the latter case revealed deposition of IgG-kappa in the corneal biopsy. Figure 1 is an image of the corneal deposits in a patient with monoclonal gammopathy and crystalline keratopathy. For simplicity we are using the term “crystalline” but these deposits, also, may be “paracrystalline.” Generally, paracrystalline states are defined as having short- and medium-range ordering in their lattice but lacking long-range ordering, at least in one direction; the most common methods of characterization of crystalline states are X-ray diffraction or cryoelectron microscopy. Figure 2 shows immunoglobulin deposits in the posterior stroma adjacent to Descemet membrane in a case of crystallizing keratopathy.^7^ Figure 3A displays the same area of the cornea stained for immunoglobulin, and Figure 3B shows electron-dense extracellular corneal crystals uncovered by transmission electron microscopy.^7^

The frequency of crystalline keratopathy in patients with essential monoclonal gammopathy is not known, but it is very uncommon. In a study of all types of monoclonal gammopathies, one case of corneal crystals was observed among 100 patients studied (patient with amyloidosis), of whom 23 patients had essential monoclonal gammopathy.^8^ Notably, bilateral crystalline corneal deposits have been the initial manifestation of myeloma.^9^

Crystalline keratopathy usually occurs as a result of crystal formation of monoclonal IgG-kappa within the cornea.^6^,^7^,^9^–^11^ The immunoglobulin depositions may appear as white, yellow, gray, or polychromatic opacities, which may show iridescence by slit lamp examination.^7^,^12^–^16^ They may be discrete or diffuse fine crystals, or dense and irregular ones.^15^ All corneal layers can be involved; stromal involvement centrally or peripherally is common. The crystals may also involve the conjunctiva and, in small quantities, the ciliary processes and choroid.^15^ The shape of the crystals may vary; the most common shape observed by transmission electron microscopy is the parallel band shape, usually exhibiting a linear internal periodicity of approximately 10 nm.^7^,^13^–^20^ The crystalline deposition, although usually extracellular, may be present in keratocytes, presumably as a result of endocytosis or phagocytosis of immunoglobulin.^7^,^17^,^19^,^20^

The most frequent type of monoclonal protein present in cases of crystalline keratopathy is IgG-kappa; this could reflect that IgG monoclonal gammopathy is the most prevalent isotype and that in 70% of those cases the monoclonal IgG is composed of kappa (rather than lambda) light chains. However, the relative prevalence of a monoclonal IgG isotype and monoclonal kappa light chains in cases of crystalline keratopathy is striking (Table 1) and suggests this isotype, especially with mutated kappa light chains, is prone to corneal crystal formation, as is the case of the IgG-kappa crystal-induced Fanconi renal syndrome, observed in monoclonal gammopathies.^21^ We are not aware of studies of a concordance between IgG-kappa-induced crystalline keratopathy and Fanconi renal syndrome with IgG-kappa-induced crystal deposition in proximal tubular cells, although one case describing both is included in this report. The cases reported of crystalline keratopathy associated with essential monoclonal gammopathy are shown in Table 1. In several cases reported associated with myeloma, the case histories indicate that the corneal disease preceded the myeloma, sometimes by years, presumably during a period of essential monoclonal gammopathy.^15^,^17^,^22^ Crystalline keratopathy has led to the diagnosis of essential monoclonal gammopathy.^23^

**Table 1:** Cases of Ocular Pathology Associated with Essential Monoclonal Gammopathy.

Citation/Year of Report	Age	Sex (M/F)	Ig Isotype	Other Tissue Involvement	Treatment	Outcome	Notes
**Crystalline Keratopathy**							
12/1959	55	F	IgG (light chain type not determined)	Iritis, conjunctivitis	None at the time of diagnosis		Five years after diagnosis of keratopathy, myeloma developed with crystals in myeloma cells
7/1979	74	F	IgG-kappa	No	Keratoplasty and cataract extraction	Not reported	Posterior corneal stroma contained parallel structures with periodicity of 10–11 nm
17/1980	57	M	IgG-kappa	Iritis	Not reported	Not reported	History of Hodgkin lymphoma treated with radiation
14/1980	39	F	IgG-kappa	Tearing, photophobia	Not reported	Not reported	IgGk deposits in corneal stroma and Bowman membrane. Electron micrograph showed parallel rod-like structures with periodicity of 10 nm
23/1985	65	F	IgA-kappa	Macular drusen	Not reported	Not reported	Slit lamp examination showed needle-like refractile, coarse crystals in mid-deep corneal stroma, bilaterally
16/1988	50	F	IgG-kappa	Uveitis	Corneal graft and posterior chamber intraocular lens insertion	Recurrence of crystalline keratopathy by 4 months	TEM revealed paracrystals with periodicity of 16 nm
25/1989	60	M	IgG-kappa (essential cryo-immunoglobulin)	No	Superficial keratectomy	Improved visual acuity	Crystallization at the corneal subepithelium in this case thought to be related to that site being the coldest in the body (~32°C)
28/1993	62	F	IgG-kappa	No	Not reported	Not reported	Crystal deposits by TEM were all extracellular, located between keratocytes
28/1993	65	M	IgG-kappa	No	Not reported	Not reported	Majority of crystals by TEM were intracellular in stromal keratocytes
29/1994	62	M	IgG-kappa	No	Penetrating keratoplasty	Not reported	IgGk deposits in cornea; TEM showed intracytoplasmic Ig in keratocytes with 10 nm periodicity
20/1996	52	F	IgG-kappa	Photophobia and foreign body sensation	Not reported	Not reported	TEM of corneal biopsy found extracellular rectangular and arcuate crystalloids with 10 nm periodicity
19/1999	55	F	IgG-kappa	No	Penetrating keratoplasty, open-sky extracapsular cataract extraction, posterior chamber intraocular lens implantation	Not reported	Decreasing visual acuity led to diagnosis crystalline keratopathy and, subsequently, of IgG-kappa essential monoclonal gammopathy. IgG-kappa-positive deposits in corneal button removed at keratoplasty; TEM showed numerous bundles of electron dense deposits of filaments with a periodicity of 10–13 nm in the anterior stroma of the cornea. Some deposits were phagocytosed by keratocytes
18/2011	62	F	IgG-kappa	No	Three penetrating keratoplasty surgeries, right-sided: at diagnosis, then 9 years and 12 years later for recurrent loss of visual acuity in the right eye	Improvement for several months post-keratoplasty followed by gradual decrease in visual acuity over years	Left eye affected but to a lesser degree than right eye; no follow-up reported after 3rd keratoplasty
**Crystal-storing Histiocytosis Involving the Eye**							
35/1990	64	F	IgA-kappa	No	Not described	Not described	Slit lamp exam revealed bilateral superficial corneal and conjunctival crystals. Diagnosed by conjunctival biopsy
10/1993	62	F	IgG-kappa	Crystalline keratopathy	4 cycles of bortezomib-based chemotherapy followed by autologous stem cell transplantation	18 months following autologous stem cell transplantation, patient in complete hematologic, renal, and ophthalmologic remission	Melphalan conditioning for auto-transplant. At 3 months post-transplant, orbital mass had significantly decreased, Fanconi syndrome resolved, and there was no detectable paraproteinemia
34/2009	66	M	IgG-lambda	Crystalline keratopathy	Not described	Not described	Right-sided keratoplasty for crystalline keratopathy 1 year prior to diagnosis of CSH. MRI revealed enhancement of retrobulbar fat and thickening of extraocular muscles; biopsy revealed CSH
**Corneal Deposition of Immunoglobulin-bound Copper**							
42/1967	69	F	IgG	No	Not described	Not described	Myeloma diagnosed 1 year after slit lamp findings of greenish-blue granular discoloration of corneal epithelium and Descemet membrane. Serum copper 13 to 24 × values found in 11 other unaffected myeloma patients tested. Serum ceruloplasmin was normal. Mild increase in urinary copper
44/1975	41	F	IgG-lambda	No	Not described	Not described	3 years before diagnosis of myeloma, patient had diagnosis of “cataracts” despite 20/20 vision. Soon thereafter cornea found to have dense brown staining reminiscent of Kayser–Fleischer type corneal rings. Serum copper 7 to 11 × normal serum value. Serum ceruloplasmin normal
39/1983	60	M	IgG-lambda	Not reported	Intracapsular cataract extraction and insertion of Binkhorst four-loop lens	Not reported	Concurrent diagnosis of lung carcinoma. Received radiation to lung. Slit lamp examination showed diffuse greenish-yellow discoloration of cornea. Serum copper 7 × normal serum value. Serum ceruloplasmin normal
41/1996	65	M	IgG-kappa	Copper depositions in anterior and posterior lens surface and appearance of sunflower cataract	Zinc gluconate	No change in serum copper	Bilateral blurred vision. Slit lamp examination revealed golden-brown deposits diffusely distributed centrally in cornea. Serum copper 12 × normal serum value. Serum ceruloplasmin normal. Special studies showed binding of copper to serum monoclonal IgG by two methods
40/2005	49	F	IgG-lambda	No	No	N/a	Patient asymptomatic. Slit lamp examination showed golden-brown metallic dust-like particles in Descemet membrane bilaterally. Serum copper 3 × normal. Serum ceruloplasmin normal
38/2014	46	F	IgG-lambda	No	Descemet-stripping endothelial keratoplasty and cataract extraction with intraocular lens implant of left eye	Complete return of normal visual acuity (20/20)	Posterior layer of both corneas had a confluent tan color with only a narrow rim of clear cornea peripherally. Serum copper 12 × normal. Serum ceruloplasmin normal
**Maculopathy**							
47/2013	37	F	Not specified	No	Prednisone with inability to wean until rituximab was initiated	Marked and rapid improvement in inflammation and visual acuity with prednisone but recurrence with taper. Resolution of ocular findings without recurrence at 9 months once rituximab therapy was initiated	Bilateral serous macular detachments, iritis, vitritis. Details of how frequently and at what dose rituximab treatment and maintenance treatment were administered are not provided

CSH, crystal-storing histiocytosis; F, female; M, male; MRI, magnetic resonance imaging; TEM, transmission electron microscopy.

Patients with crystalline keratopathy may present with bilateral loss of visual acuity,^19^,^24^,^25^ unilateral vision loss,^17^,^25^ or they may be asymptomatic.^17^,^24^,^25^ Loss of vision may occur gradually and progress over years.^10^,^18^,^25^ The diagnosis of crystalline keratopathy is made when slit lamp examination and, sometimes, confocal microscopy reveal the deposition of crystals throughout the corneal stroma, usually bilaterally.^9^–^11^,^13^,^14^,^17^–^19^,^24^–^26^ Crystalline keratopathy may be discovered coincidentally with other ocular pathology caused by an underlying monoclonal gammopathy, such as palpebral ecchymoses due to vascular fragility secondary to amyloid deposition^11^ and as crystal-storing histiocytosis, a pathologic entity that is discussed below.^10^

The specific process of crystal deposition in the cornea is uncertain.^10^ Immunoglobulins are found, normally, in the major ocular structures except for the lens, but at a tissue concentration somewhat less than that of plasma. The cornea has the highest concentration of IgG per gram of tissue, and most is found in the corneal stroma.^27^ It has been postulated, based on immunoelectron microscopic analysis of corneal tissue from two patients with essential monoclonal gammopathy and crystalline keratopathy, that immunoglobulin may be delivered to the cornea via the limbal microvasculature.^20^,^27^,^28^ In patients with crystalline keratopathy in whom myeloma is evident, the marrow myeloma cells may show crystalline inclusions identical to those observed in the cornea by both light and electron microscopy.^5^,^12^,^13^

There is little information regarding the natural history of crystalline keratopathy and its treatment.^5^ Some cases are associated with no or minimal visual symptoms and may require no therapy. In cases with severe and irreversible corneal involvement, penetrating keratoplasty can be performed, but corneal crystal deposition can recur if the monoclonal immunoglobulin remains present.^7^,^18^,^19^,^25^,^26^,^29^ Treatment targeting the underlying monoclonal gammopathy usually improves ocular symptoms.^24^,^30^ This observation was made because of the need to treat patients with severe symptomatic monoclonal gammopathies, such as those with myeloma or with concomitant Fanconi renal syndrome.

## CRYSTAL-STORING HISTIOCYTOSIS INVOLVING THE EYE

Accumulation of monoclonal immunoglobulin crystals, predominantly of a kappa light chain type, within lysosomes of macrophages within the organs of the mononuclear phagocyte system, e.g. marrow, liver, spleen, and lymph nodes, and, in some cases, other extramedullary tissues characterizes the disorder crystal-storing histiocytosis (CSH).^10^,^31^,^32^ The morphology of the histiocytes may appear superficially similar to Gaucher cells when examined by light microscopy.^32^,^33^

We are aware of nine cases of ocular CSH in patients with monoclonal gammopathies.^10^,^34^,^35^ The monoclonal gammopathies that have been associated with CSH are usually myeloma, solitary plasmacytoma, and lymphoplasmacytic lymphoma.^32^ Rare cases of essential monoclonal gammopathy have been accompanied by this disorder.^10^,^32^,^34^–^37^

Crystal-storing histiocytosis affecting the eye associated with essential monoclonal gammopathy has been reported to involve the conjunctiva and to infiltrate the orbital fat and extraocular muscles, sometimes causing invasive masses.^10^,^34^ Crystal-storing histiocytosis usually is found in more than one organ in a patient, especially the principal organs of the mononuclear phagocyte system. One patient with an IgG-kappa essential monoclonal gammopathy developed an orbital mass due to CSH and also had crystal-laden macrophages invading the renal interstitium and proximal tubules, causing immunoglobulin-associated Fanconi renal syndrome.^10^ This patient, also, had crystalline keratopathy.

**Figure 4:**
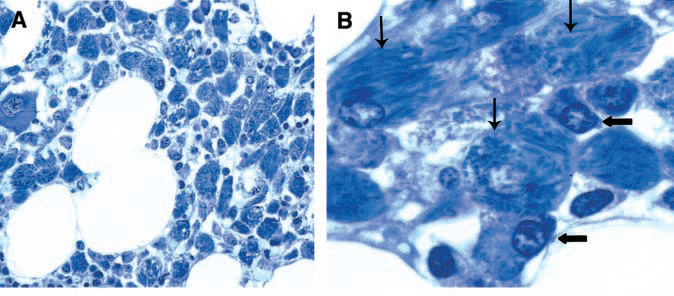
Crystal-storing Histiocytosis. Patient with Essential Monoclonal Gammopathy (IgA-kappa Type). Marrow Biopsy Treated with Giemsa Stain (A) The marrow shows a diffuse infiltrate of histiocytes (larger cells) with crystalloid cytoplasmic inclusions (original magnification ×1,600). (B) Plasma cells were infrequent. In this field two are shown by horizontal arrows. Three histiocytes with cytoplasmic crystals are indicated by the vertical arrows (original magnification ×4,000). (Used from reference 32 with permission of the American Society of Hematology.)

The diagnosis of CSH is made by biopsy of affected tissue and subsequent histopathological analysis.^10^,^32^,^34^ Overproduction of monoclonal kappa light chains in concert with their conformational change presumably leads to their crystallization in the lysosomes of histiocytes (Figure 4).^34^ One hypothesis is that mutations of the DNA sequence encoding the variable region of the involved light chain may lead to exposure of hydrophobic regions of the resultant protein, making it resistant to enzymatic degradation by macrophages.^31^,^32^ The reason for tropism of CSH for orbital or periorbital structures is unclear.

There is a paucity of information regarding prognosis and optimal treatments of CSH-related orbitopathy. However, in the case of essential monoclonal gammopathy in which both ocular and renal manifestations of CSH were present, in addition to corneal crystalline keratopathy, initiation of myeloma-type therapy and subsequent autologous hematopoietic stem cell transplantation resulted in complete hematological, ophthalmological, and renal remissions at 18 months post-transplant.^10^

## CORNEAL DEPOSITION OF MONOCLONAL IMMUNOGLOBULIN-BOUND COPPER

Deposition of immunoglobulin with a strong affinity for copper in the cornea, resulting in accumulation of copper in that site, has been associated with monoclonal gammopathy and can cause impairment of visual acuity. The underlying systemic disorders were usually IgG-lambda and less often IgG-kappa essential monoclonal gammopathy, chronic lymphocytic leukemia with monoclonal IgG (light chain not specified), IgG-kappa-type myeloma, and IgG myeloma (light chain not specified).^38^–^42^

**Figure 5:**
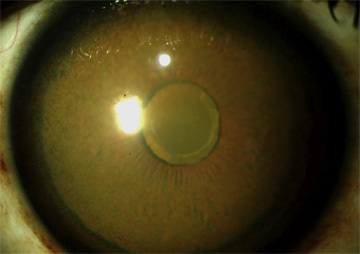
Appearance of the Left Cornea in a Patient with Essential Monoclonal Gammopathy (IgG-lambda) Brownish-green discoloration was evident in Descemet membrane, sparing the peripheral 1–2 mm. The anterior lens capsule is discolored. Light reflection off the anterior lens capsule is evident. The serum copper was 1,773 μg/dL (normal 70–175), and serum ceruloplasmin was normal. (Used from reference 38 with the permission of Elsevier.)

Patients may present with gradually progressive visual changes. The diagnosis should be suspected upon visualization by slit lamp examination and confocal microscopic imaging of a central, circular, yellow-brown discoloration of each cornea secondary to pigmentation in Descemet membrane.^43^ The copper deposition may extend to the lens capsule. This pattern of staining can be distinguished from the typical ocular changes seen in Wilson disease, in which there are usually annular corneal yellow-brown opacities involving only the periphery of Descemet membrane, rarely extending more than a few millimeters centrally (Kayser–Fleischer ring) (Figure 5).^38^,^43^ If corneal discoloration is suspicious for a pattern consistent with copper deposition, measurement of serum proteins to assess the presence of a monoclonal gammopathy should be performed.

Corneal copper deposits due to Wilson disease may be confirmed utilizing biochemical data; Wilson disease is usually characterized by decreased serum ceruloplasmin and elevated 24-hour urine copper excretion. Patients with a high-affinity, copper-binding immunoglobulin have extraordinarily high serum copper levels, sometimes 10- to 25-fold the normal serum copper concentration, normal to mildly elevated urinary copper, and normal serum ceruloplasmin levels.^42^,^44^ Immunoglobulins are non-metal-containing glycoproteins. In cases of corneal copper deposition associated with monoclonal gammopathy (or myeloma), copper migrates with the gamma fraction of globulins in electrophoretic studies, and elution studies on diethylethylaminoethyl (DEAE)-cellulose columns confirm IgG copper-binding to the monoclonal immunoglobulin. In cases so studied, a radioactive copper isotope was bound to the IgG fraction of serum, specifically.^41^ The structural explanation for high-affinity copper-binding IgG in rare cases of monoclonal gammopathy is unknown. Analysis has not confirmed an increase in copper-binding amino acid residues when compared to monoclonal IgG in which copper-binding is not a feature.^41^ In one report, energy-dispersive X-ray microanalysis of the lens capsule tissue revealed copper deposition; immunoperoxidase staining for IgG-lambda performed on the same tissue showed linear uptake.^38^ Despite very high serum levels of copper, evidence of other cellular toxicity is absent because the monoclonal immunoglobulin binds copper more tightly than ceruloplasmin, preventing cellular injury by free copper. The reason for selective deposition of copper in the Descemet membrane or the lens capsule is not known. It is speculated that there are factors unique to the anterior chamber that may have an effect favoring localization of the copper–immunoglobulin complex.^38^

Given the rarity of this condition, optimal management strategies are not defined, but presumably the approach depends upon the underlying cause of the monoclonal gammopathy and whether or not vision is affected. In the case of myeloma that is associated with copper-binding immunoglobulin and corneal copper deposition, appropriate cytotoxic therapy for myeloma may slow or stop further discoloration of ocular basement membranes and, presumably, further impairment of vision. Attempts at treating corneal copper deposits associated with an underlying essential monoclonal gammopathy have been unsuccessful. Descemet membrane-stripping endothelial keratoplasty or full-thickness keratoplasty can provide temporary relief of visual symptoms, but they ultimately recur if there is persistence of the monoclonal immunoglobulin. Cataract extraction with removal of the anterior lens capsule and subsequent posterior capsulotomy can be performed to resolve lenticular changes and capsular discoloration permanently.^38^

## MACULOPATHY

Maculopathy with retinal detachment has uncommonly been described in association with monoclonal gammopathy. In a review of 33 patients with a monoclonal-protein-associated maculopathy, 20 of the patients had Waldenström macroglobulinemia, 8 had myeloma, 2 had light chain deposition disease, 1 had hypergammaglobulinemia associated with systemic lupus erythematosus, 1 had polyclonal hypergammaglobulinemia without a known underlying cause, and 1 patient had polyneuropathy organomegaly endocrinopathy M-protein and skin abnormalities syndrome (POEMS).^45^ Nine of the patients had unilateral involvement.

Systemic therapies differed on the basis of the type of underlying clonal disorder, and ocular therapies also varied among patients. Over the course of a 7-month follow-up period, the maculopathy resolved partially or fully in 17 patients and was stable or worsened in 14 patients.^45^

The frequency of maculopathy among patients with monoclonal gammopathies has not been reported. As noted above, it has most frequently been reported in patients who have a circulating monoclonal IgM due to Waldenström macroglobulinemia.^45^ Patients with serous macular detachment typically present with progressive vision loss over the course of weeks to months.^46^ Maculopathy and serous macular detachment associated with monoclonal gammopathies may be unilateral or bilateral.^45^,^46^ Diagnosis is made via dilated fundoscopic examination.^46^ If serous macular detachment is suspected, optical coherence tomography and fluorescein angiography can be useful in delineating the injury.

In a report of a 37-year-old woman with monoclonal gammopathy (type not specified)—notably the only report that we found in the literature of a maculopathy associated with essential monoclonal gammopathy (rather than other progressive monoclonal gammopathies)—associated with bilateral iritis, vitritis, and serous macular detachments, treatment with oral and topical glucocorticoid therapy resulted in rapid improvement of these findings with full return of visual acuity. Tapering of prednisone led to recurrence of ocular abnormalities and vision loss that resolved with its resumption; rituximab therapy was then initiated and maintained, which allowed the withdrawal of glucocorticoids. Given this response to treatment, the authors of this report hypothesized that circulating monoclonal immunoglobulin may act as an autoantibody against a specific retinal structure. However, there is no additional published evidence in support of retinal autoantibodies as the etiology for monoclonal gammopathy associated maculopathy. There was no immunohistochemical staining done on biopsied ocular tissues to analyze for the presence of immunoglobulin.^47^

The pathogenesis of maculopathy and serous macular detachment associated with monoclonal gammopathy currently remains unknown. In 1965, protein deposits were noted in two patients with myeloma within the subretinal space and uvea on post-mortem analysis. These protein deposits were not characterized.^48^ Angiographic studies have not shown significant leakage of retinal and choroidal vasculature in the cases of monoclonal gammopathy-associated maculopathy.^45^,^46^,^49^ There are reports of patients with Waldenström macroglobulinemia^50^,^51^ and also with myeloma^52^ in which there was extracellular deposition of the monoclonal immunoglobulin in the retinal tissue and the subretinal space as demonstrated by immunofluorescence or immunoelectrophoresis techniques. It has, therefore, been suggested that, in the maculopathy associated with monoclonal gammopathy, extravasation of the immunoglobulin primarily occurs from the retinal vessels, with subsequent deposition in the subretinal space; an increase in oncotic pressure ensues and can cause serous macular detachments.^45^,^53^

Given the small number of cases, it is not possible to make any conclusions regarding the prognosis with regard to visual acuity of patients who have maculopathy associated with monoclonal gammopathies, but it does seem to be highly variable.^45^ In Waldenström macroglobulinemia, some patients may have loss of visual acuity due to elevated IgM and the effects of the hyperviscosity syndrome and resulting ischemia on the eye, which may improve with plasmapheresis; but this procedure is less useful for treating associated maculopathy.^45^ For patients who have symptomatic maculopathy, initiation of chemotherapy may be indicated. In a patient with smoldering myeloma who had associated serous macular detachments, treatment with bortezomib and dexamethasone resulted in resolution of maculopathy and serous macular detachments, and subsequent restoration of visual acuity.^54^

## CONCLUSION

Three principal types of ophthalmic injury, crystalline keratopathy, crystal-storing histiocytosis with orbitopathy, and corneal copper deposition involving Descemet membrane and the lens capsule, can occur as manifestations of uncommon physicochemical features of the immunoglobulin in cases of essential monoclonal gammopathy (and other progressive monoclonal gammopathies). A fourth, rarer syndrome, maculopathy with serous retinal detachment, has an unknown pathogenesis. If there are concomitant tissue effects, such as Fanconi renal disease or findings of progressive myeloma, justifying intensive treatment of the underlying monoclonal gammopathy, and treatment is successful, the ocular pathology usually ceases to progress, improves, or resolves. Given the infrequency of these types of ophthalmic syndromes secondary to monoclonal gammopathy, there are no specific treatment guidelines. However, case reports support the concept that in some situations ocular surgical interventions and/or therapeutic targeting of the underlying monoclonal gammopathy can sometimes improve or restore visual acuity. Visual symptoms in persons with monoclonal gammopathy should be evaluated promptly by an ophthalmologist.

## Abbreviations

CSH: crystal-storing histiocytosis
DEAE: diethylethylaminoethyl
Ig: immunoglobulin
MRI: magnetic resonance imaging
TEM: transmission electron microscopy.
